# Zona auriculaire compliqué d'un syndrome vestibulaire invalidant

**DOI:** 10.11604/pamj.2015.20.179.6128

**Published:** 2015-02-26

**Authors:** Madiha Mahfoudhi, Khamassi Khaled

**Affiliations:** 1 Service de médecine interne A Hôpital Charles Nicolle, Tunis, Tunisie; 2 Service ORL, Hôpital Charles Nicolle, Tunis, Tunisie

**Keywords:** Zona, diabète, syndrome vestibulaire, surdité, Zona, diabète, syndrome vestibulaire, surdité, Zona, diabetes, vestibular syndrome, deafness

## Image en medicine

**Figure 1:**
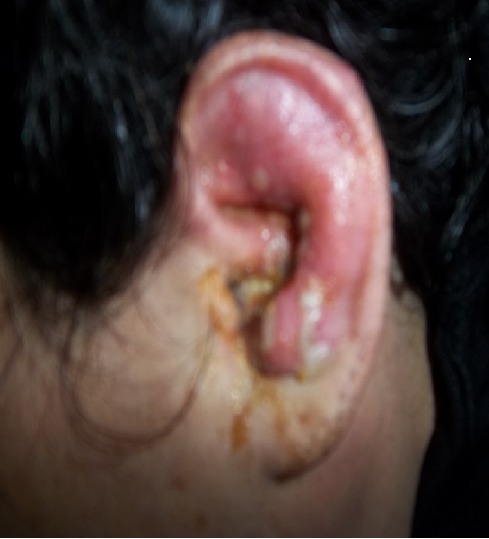
Éruption érythémateuse et vésiculeuse de la conque et du pavillon gauche

Le zona correspond à une manifestation de récurrence du virus Varicelle-Zona (VVZ). La forme auriculaire complète est peu fréquente et peut entrainer des séquelles graves en cas de retard diagnostique et thérapeutique. Femme âgée de 35 ans diabétique de type I, hospitalisée pour des crises vertigineuses intenses et récurrentes associées à des vomissements évoluant depuis deux jours. Elle présentait une otalgie, une hypoacousie et une otorrhée gauche. L'examen de l'oreille gauche a objectivé une tuméfaction inflammatoire du pavillon, des vésicules à contenu jaune citrin au niveau de la conque (Figure 1), un conduit auditif externe inflammatoire et un tympan congestif. Un syndrome vestibulaire a été objectivé avec une station debout impossible. Le reste de l'examen notamment neurologique était sans anomalies. Elle avait un syndrome inflammatoire biologique et un diabète déséquilibré. L'audiométrie tonale a révélé une surdité de perception de 60 dB à gauche. L’épreuve calorique a confirmé un déficit vestibulaire aigue à gauche. Le traitement a associé Aciclovir, corticoïdes, antiémétiques, antivertigineux, soins locaux quotidiens et équilibration de son diabète. L’évolution était marquée par une amélioration clinique et biologique. Elle a gardé une surdité de perception gauche séquellaire de 60 dB.

